# Design and simulation of a high performance Ag_3_CuS_2_ jalpaite-based photodetector

**DOI:** 10.1016/j.heliyon.2024.e32247

**Published:** 2024-05-31

**Authors:** Sheikh Noman Shiddique, Md. Islahur Rahman Ebon, Md. Alamin Hossain Pappu, Md. Choyon Islam, Jaker Hossain

**Affiliations:** Solar Energy Laboratory, Department of Electrical and Electronic Engineering, University of Rajshahi, Rajshahi, 6205, Bangladesh

**Keywords:** Ag_3_CuS_2_ jalpaite, Responsivity, Detectivity, Photodetector, Near-IR, SCAPS-1D

## Abstract

This work provides a comprehensive investigation by using simulations and performance analysis of a high performance and narrowband Ag_3_CuS_2_ photodetector (PD) that operates in the near-infrared (NIR) region and is built using WS_2_ and BaSi_2_ semiconductors. Across its operational wavelength range, a comprehensive assessment of the device's electrical and optical properties such as photocurrent, open-circuit voltage, quantum efficiency, responsivity and detectivity is methodically carried out. Furthermore, a thorough investigation has been conducted into the impact of many parameters, including width, carrier density and defects of various layers. Also, the intricate interactions between WS_2_/Ag_3_CuS_2_ and Ag_3_CuS_2_/BaSi_2_ interface properties of the photodetector are explored. The Ag_3_CuS_2_-based PD remarkably produces the best outcomes with an open-circuit voltage of 0.74 V, current of 43.79 mA/cm^2^, responsivity of 0.79 AW^-1^ and detectivity of 4.73 × 10^14^ Jones and over 90 % QE in the NIR range for the Ag_3_CuS_2_ PD. The results showcase this jalpaite material as a promising one in the field of PD.

## Highlights

• High performance Ag_3_CuS_2_ Jalpaite thin film photodetector has been modeled and computed.
• In the design, WS_2_ and BaSi_2_ have been used as window and BSF, respectively.
• The device shows of a voltage of 0.74 V and current of 43.79 mA/cm^2^, respectively.
• Ag_3_CuS_2_ PD provides a responsivity of 0.79 AW^-1^ and detectivity of 4.73 × 10^14^ Jones.
• The designed Ag_3_CuS_2_ PD proves itself as a promising one in the field of PD.

## 1 Introduction

An essential part of contemporary technology, a photodetector is a device that transforms light into electrical signals. This indispensable instrument finds extensive usage throughout diverse sectors from sensing technology to communications [1]. Photodetectors are essential for allowing information to be transmitted across optical fibers and enabling effective communication networks because they use the power of photons. Photodetectors, as the portal to the world of light, are still driving innovation and reshaping the electronics and communications scene of today [2].

Because of the hefty propagation and lower abatement properties of near-infrared (NIR) light, NIR photodetectors that can convert light energy into electrical signals are currently attracting a lot of interests for use in security, biomedical imaging, night vision and optical fibers communications [[1], [2], [3], [4], [5], [6]]. Traditionally, NIR PDs are made employing InGaAs and Ge-based devices with a cut-off wavelength greater than 1.5 μm. Because of having low cut-off wavelength of 1.1 μm, silicon (Si)-based photodetectors exhibit low external quantum efficiency and are difficult to employ for NIR wavelength detection [7,8]. In addition, CsSn_0.5_Ge_0.5_I_3_ perovskite PD has been showcased with a responsivity of 0.54 A/W and detectivity of 3.3 × 10^13^ Jones [9]. On the other hand, PDs made of Ge semiconductor experience an elevated reverse or dark current (∼10 mA/cm^2^) which is very higher in magnitude than that of the InGaAs PDs (∼0.5 μA/cm^2^). Therefore, low signal-to-noise ratio (S/N) and responsiveness results are achieved from this Ge-based PD structure [[10], [11], [12]]. However, the problems arise due to the expensive production methods associated with InGaAs-based photodetectors [13,14].

Recently, near-infrared photodetectors (NIR-PDs) have been produced by a technique that involves spin coated colloidal quantum dots (QDs) [15]. But the gadget had drawbacks, such as a narrow 3-dB bandwidth of only 18 Hz and a high working voltage of 40 V. Their wider practical utility in these sectors is hindered by these limitations, which significantly limit their viability in imaging and communication applications that require high-speed and low-power photodetectors [16]. Present generation of widely used infrared photodetectors mostly make use of narrow-band-gap compounds like InSb, HgCdTe and PbS QDs [[17], [18], [19]]. However, there are a number of disadvantages that these devices frequently face, such as toxicity to the environment, complex production procedures, high costs and significant response times [20]. Moreover, in the present conversations, there is still disagreement and doubt surrounding the assessment of Quantum dot infrared photodetectors (QDIP's) and Quantum wire infrared photodetectors (QRIP's) capabilities [21]. These drawbacks prevent them from being widely used, which calls for improvements in photo-detection technologies to solve these issues and make infrared sensing more effective and widely available for a range of applications.

Herein, the more straightforward ternary compound Ag_3_CuS_2_ (jalpaite) has been used as an absorber material for high performance NIR photoactive layer. Ag_3_CuS_2_ belongs to *I*4_1_/*amd* space group and tetragonal shape with unit cell parameters of a = 8.6705 Å and c = 11.7573 Å [22]. Ag_3_CuS_2_ has previously been demonstrated to exhibit photocatalytic behavior when combined with Ag_2_S and Ag [23]. But the recent works on solar cells that use it as an absorber layer has made it immediately interesting [24,25]. The key role of an absorber is to generate electron-hole pairs which is perfectly done by this jalpaite material [26]. Since Breithaupt initially identified Ag_3_CuS_2_ as the mineral jalpaite in 1858, mineralogists have been interested in this compound due to its intriguing characteristics as well as its significance as a noble metal resource [[27], [28], [29]]. Ag_3_CuS_2_'s prospective uses as copper-selective electrodes have been documented in earlier research [28,29]. This Ag_3_CuS_2_ compound has been suggested as a potential photon absorbing material for photovoltaic cells and other optoelectronic devices because of its (i) higher light absorption capability in the visible regions, (ii) lower toxicity, and (iii) readily available and affordable raw materials [24]. Although, some experimental works have been conducted on Ag_3_CuS_2_ finding the crystal structure, Seebeck coefficient, electrical conductivity, thermal conductivity, effective masses, direct bandgaps, carrier mobilities indicating its application in different fields such as thermoelectric and photovoltaic [[30], [31], [32]]. However, no experimental work has been done as a photodetector. In this work, Ag_3_CuS_2_ compound has been chosen as the absorber material in the photodetector (PD) application because of its aforementioned benefits.

Native n-type semiconducting tungsten disulfide (WS_2_) belongs to the space group of C_2/m_ and displays a hexagonal shape. The lattice parameters of this compound are a = 12.8417 Å, b = 3.2177 Å and c = 5.6912 Å, separately and it shows a greater stability at thin condition which enhances thermal conductivity [33]. WS_2_ includes strong mobility of carriers and superior electrical conductivity in the range of 10^−3^ Ω^−1^ cm^−1^ [34,35]. Comparing WS_2_ to other transition metal dichalcogenide (TMDC) materials, it is less hazardous, less expensive and more prevalent in the Earth's crust [36]. Its adjustable bandgap is a crucial feature that is rarely discussed. Depending on how it is made, WS_2_ can have a low indirect bandgap (<1.5 eV) or a large direct bandgap (>2 eV) [37]. Its significantly larger W atom size allows it to modify its structural characteristics according to the use for which it is intended [37,38]. WS_2_ has been selected as the window layer for Ag_3_CuS_2_ based PD due to its distinct optoelectronic and electrochemical characteristics.

Barium silicide (BaSi_2_) which is made from barium (Ba) and silicon (Si) has some interesting properties that bring out its attention to be used as a back surface layer in Ag_3_CuS_2_ based PD. Barium is a soft silvery-gray colored element with an atomic number of 56 [39]. BaSi_2_ belongs to the P_nma_ space group and orthorhombic structure with lattice constants of a = 8.942 Å, b = 6.733 Å and c = 11.555 Å, separately [40]. BaSi_2_ has found applications in thin film solar cell (TFSC) due to its good chemical stability and wide bandgap ranging from 1.1 to 1.35 eV [41]. Since Ba and Si are readily available on Earth, adding a thin layer of BaSi_2_ might significantly lower the cost of producing Ag_3_CuS_2_ PD. Approximately 30–40 times more absorption occurs in BaSi_2_ than in single-crystalline silicon (c-Si) [42,43]. BaSi_2_ is thought to be the ideal absorber material for solar cells based on both experimental and theoretical investigations [39,[41], [42], [43]].

This article reports Ag_3_CuS_2_ based PD with WS_2_ and BaSi_2_ window and BSF layer, respectively. The photo-sensing performance of the device has been numerically probed in details by considering different parameters of the constituent layers showcasing it's capability as a PD device.

## 2 Designing of device and computational approach

**Fig. 1:**
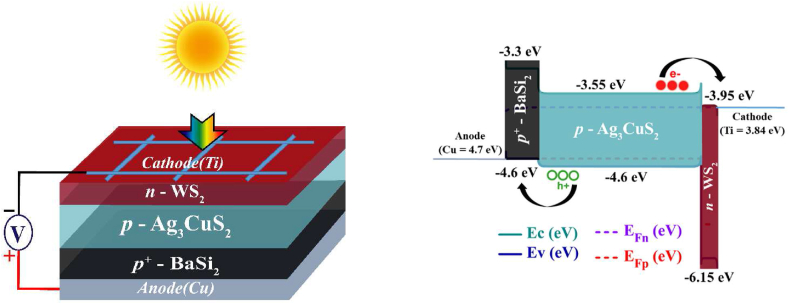
**(a)** Illustrative block and (b) band diagram of proposed *n*-WS_2_/*p*-Ag_3_CuS_2_/*p* ^*+*^ -BaSi_2_ PD.

Fig. 1(a) and (b) show the structure of the modeled *n*-WS_2_/*p*-Ag_3_CuS_2_/*p* ^*+*^ -BaSi_2_ photodetector with its energy band diagram, in sequence. Ag_3_CuS_2_ possesses a bandgap of 1.05 eV with electron affinity 3.55 eV that make it a suitable candidate for use as a photodetector. WS_2_ is the window layer, and BaSi_2_ is the BSF layer used in the Ag_3_CuS_2_ based PD. Both compound materials present bandgaps of 2.2 and 1.3 eV and electron affinities of 3.95 and 3.3 eV, in turn. Therefore, WS_2_ and BaSi_2_ forms effective n-p-p^+^ heterojunction with Ag_3_CuS_2_ semiconductor.

However, the interface defects may be introduced as there exists lattice-mismatch between WS_2_/Ag_3_CuS_2_ and Ag_3_CuS_2_/BaSi_2_ junctions. Consequently, reasonable amount of defects have been considered in the interfaces as in Table 1. The Ti and Cu front and back contacts with respective work functions of 3.84 and 4.7 eV are used to collect carriers efficiently. Appropriate alignment and effective carrier transportation to the electrodes are the outcomes of selecting the window and BSF layers.

**Table 1:** Input parameters used for simulating Ag_3_CuS_2_ based photodetector.

Parameters	*n* -WS_2_	*p*-Ag_3_CuS_2_	*p* ^*+*^ - BaSi_2_
Width (μm)	0.1	0.8	0.2
Bandgap, E_g_ [eV]	2.2	1.05	1.3
Affinity of electrons, χ [eV]	3.95	3.55	3.3
Dielectric permittivity (relative)	13.6	10	15
Effective DOS at CB [cm^−3^]	2.5 × 10^18^	3.42 × 10^18^	2.6 × 10^19^
Effective DOS at VB [cm^−3^]	1.8 × 10^19^	7.40 × 10^18^	2 × 10^19^
Mobility of electrons, μ_n_ [cm^2^V^−1^s ^−1^]	1 × 10^2^	1 × 10^2^	2 × 10^1^
Mobility of holes, μ_p_ [cm^2^V^−1^s^−1^]	1 × 10^2^	6.6 × 10^1^	2 × 10^1^
Density of donors, N_D_ [cm^−3^]	1 × 10^18^	0	0
Density of acceptors, N_A_ [cm^−3^]	0	1 × 10^17^	1 × 10^19^
Type of defect	Single Acceptor	Neutral	Single Acceptor
Total defect density, N_t_ [cm^−3^]	1.0 × 10^14^	1.0 × 10^14^	1.0 × 10^14^

**Interface input parameters:**		
**Parameters**	***p-*Ag**_**3**_**CuS**_**2**_**/*n* -WS**_**2**_	***p*** ^**+**^ **- BaSi**_**2**_**/*p*-Ag**_**3**_**CuS**_**2**_
Defect type	neutral	neutral
Total density (1/cm^2^)	1.0 × 10^10^	1.0 × 10^10^

SCAPS-1D software is used to compute the Ag_3_CuS_2_ based PD. It is frequently used to examine different solar cells and photodetectors. The simulator may include various characteristics like bandgap, work function, doping, thickness, and defects. To find voltage, photocurrent, quantum efficiency, capacitor voltage, and C-f, SCAPS solves a set of equations. Poisson's equation, the hole and the electron continuity equations are among the equations that are involved [[44], [45], [46], [47], [48]].

(1) $${(\text{Poisson}}^{\prime }s\;\text{equation})\;\frac{\partial^{2}\Psi }{\partial x^{2}}+\frac{q}{\varepsilon }\left[p\left(x\right)-n\left(x\right)+N_{D}-N_{A}+\rho_{p}-\rho_{n}\right]=0$$

(2) $$\left(\text{Hole}\;\text{continuity}\;\text{equation}\right)\;\frac{1}{q}\frac{\partial J_{p}}{\partial x}=G_{op}-R\left(x\right)$$

(3) $$\left(\text{Electron}\;\text{continuity}\;\text{equation}\right)\;\frac{1}{\;q}\frac{\partial J_{n}}{\partial x}=-G_{op}+R\left(x\right)$$

wherein, ε, q, N_A_, N_D_, J_p_ and J_n_ denote relative dielectric constant, electronic charge, ionized acceptors, donors, current due to holes, and electrons, individually. Farther, Ψ, G_op_, R, p, n, ρ_p_, and ρ_n_ showcase the electrostatic potential, generation, recombination rate, free holes, electrons, of holes and electrons allocation, in sequence.

Effective density of states (DOS) at conduction band (CB) and effective DOS at valence band (VB) are calculated from Equations (4), (5)) by using the value of the effective mass of electrons ($m_{e}^{*})$ and holes ($m_{h}^{*})$ [49]:

(4) $$N_{v}={2\left(\frac{m_{h}^{*}KT}{2\pi \hbar^{2}}\right)}^{\frac{3}{2}}$$

(5) $$N_{c}={2\left(\frac{m_{e}^{*}KT}{2\pi \hbar^{2}}\right)}^{\frac{3}{2}}$$

However, the effective mass of electrons and holes of Ag_3_CuS_2_ are 0.265 and 0.443, respectively [50].

At the spectrum of 1.5 G AM, this photodetector come on with a power density of 100 mW/cm^2^. According to Table 1, the thermal velocity of both electrons and holes is 10^7^ (cms^−1^). The operating temperature in the simulator is set to 300 K. The parameters exerted for BaSi_2_, WS_2_ and Ag_3_CuS_2_ is acquired from literatures [39,44,50]. The sqrt (.)-E_g_ with in-built settings is used for reckoning the optical absorption. Table 1 shows the simulation parameters of Ag_3_CuS_2_ based photodetector. The photocurrent, photovoltaic, cutoff wavelength, quantum efficiency, and photon energy values are obtained from the single shot calculation. The value of responsivity has been estimated using the photon energy and QE values. The formula below is used to calculate responsivity [45]:

(6) $$R=\frac{q\eta \lambda }{\text{hc}}=\frac{J_{SC}}{P_{0}}$$

where, R= Responsivity, h = Planck's constant (6.634 × 10^−34^ J-s), c = Speed of light (3 × 10^8^ m/s), q = Electronic charge (1.6 × 10^−19^ C) and P_0_ is incident optical power.

By the value of R, the value of detectivity can be computed using the following equation [46]:

(7) $$D^{*}=\frac{R}{\sqrt{2qJ_{0}}}$$

With $J_{0}=\frac{J_{SC}}{e^{\frac{Voc}{Vt}}-1}$

Where, D = Detectivity, q = 1.602 × 10^−19^ C, V_t_ is the thermal voltage and J_0_ = Dark current.

## 3 Results and discussion

### 3.1 Ag_3_CuS_2_ based PD with & without BSF layer

**Fig. 2:**
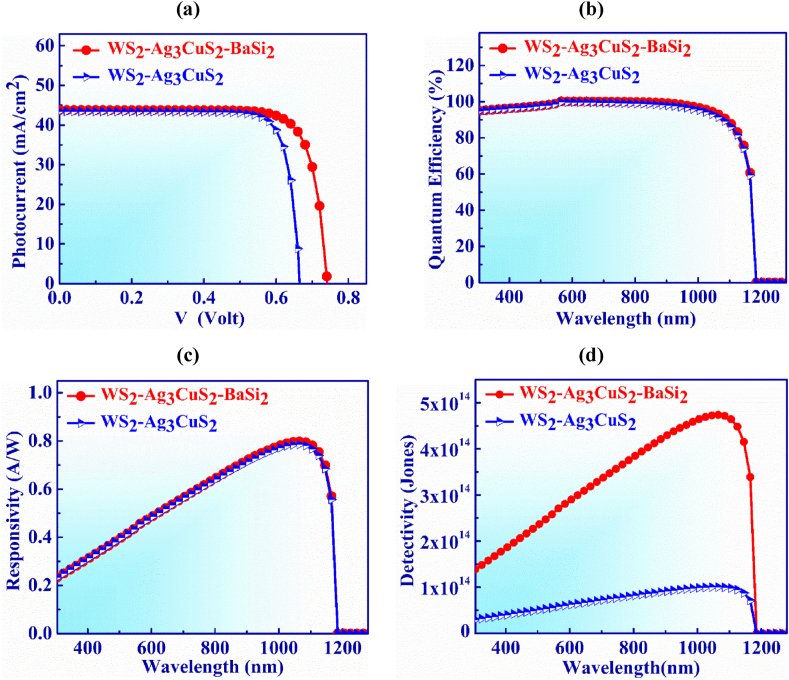
(a) Photocurrent vs. Voltage (b) quantum efficiency (QE) vs. Wavelength and (c) Responsivity vs Wavelength and (d) Detectivity vs. Wavelength characteristic curves of Ag_3_CuS_2_ based photodetector with and without BaSi_2_ BSF.

The current-voltage (J-V) curves of an Ag_3_CuS_2_-based photodetector with and without a p^+^-BaSi_2_ BSF layer are shown in Fig. 2(a). With the addition of a 200 nm thick p^+^-BaSi_2_ BSF layer between the Ag_3_CuS_2_ and the Cu back metal connection, the J_SC_ stays constant at 43.45 mA/cm^2^, but the V_OC_ increases noticeably from 0.66 to 0.74 V. Ag_3_CuS_2_/BaSi_2_ makes a p-p^+^ heterojunction interface that generates an electric field which serves as a significant obstacle to electron transport to the rear surface. This electron reflection directs the suppression of saturation current and the BSF layer curtails the recombination and magnifies the back surface potential which improves V_OC_ of the Ag_3_CuS_2_ photodetector [51,52].

Fig. 2(b) illustrates the QE vs. wavelength for Ag_3_CuS_2_ based photodetector with and without a p^+^-BaSi_2_ back surface. It is perceived that QE exhibits consistent behavior and is unaffected by BaSi_2_ BSF layer. The QE of the PD is 100 % in the wavelength range of 550–800 nm. Moreover, at wavelengths longer than 1165 nm, QE drops to zero since photon is not absorbed lower the bandgap energy of the absorber in the PD.

The responsivity exhibits consistent characteristics as seen in Fig. 2(c) just as QE does for with and without BSF layer. The greatest values of 0.79 AW^-1^ and 0.78 AW^-1^ for responsivity are displayed at 1065 nm with and without BaSi_2_ layer, respectively.

Fig. 2(d) illustrates the detectivity vs. wavelength for Ag_3_CuS_2_ photodetector with and without a p^+^-BaSi_2_ BSF layer. The detectivity increases to 4.73 × 10^14^ Jones from 1 × 10^14^ Jones when BaSi_2_ layer is put on the detector. This is due to the fact that carrier recombination falls owing to the development of built-in voltage at the Ag_3_CuS_2_/BaSi_2_ hetero-interface that enhances the V_OC_ and this causes the drop in dark current [53]. Therefore, the detectivity of the Ag_3_CuS_2_ PD increases as the dark current falls.

### 3.2 Effect of Ag_3_CuS_2_ absorber layer

#### 3.2.1 Impact of absorber width on Ag_3_CuS_2_ PD

**Fig. 3:**
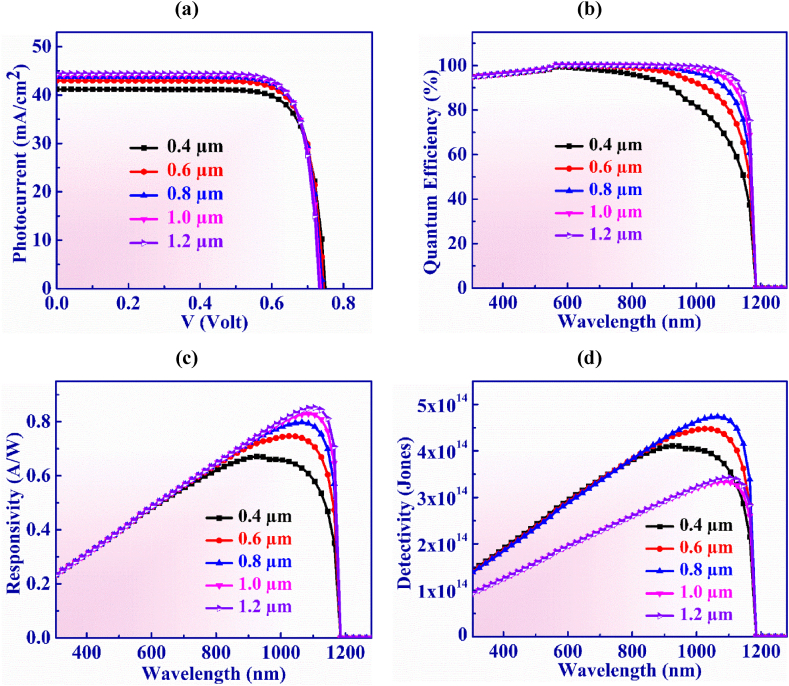
(a) Photocurrent vs. Voltage (b) QE, (c) Responsivity and (d) Detectivity characteristic curves of Ag_3_CuS_2_ photodetector with Ag_3_CuS_2_ layer thickness.

In this part, the role of the thickness of Ag_3_CuS_2_ photon absorbing layer on the functioning of Ag_3_CuS_2_ PD has been illustrated in details. The Ag_3_CuS_2_ layer's width is varied between 0.4 and 1.2 μm as shown in Fig. 3(a). In the analysis, the V_OC_ essentially stays constant at 0.74 V. However, further improvement in thickness could result in a higher recombination current and also the effective conveyance of the majority of photogenerated electron-hole pairs to their corresponding electrodes, there is a diminishing impact on the open-circuit voltage (V_OC_) [54,55]. Nonetheless, J_SC_ rises from 41.18 to 44.43 mA/cm^2^ in the thickness range under investigation. Whenever the absorber layer elongates, extra photons are get absorbed that clearly enhances the J_SC_ [56].

QE versus light wavelength for Ag_3_CuS_2_ layer breadths ranging from 0.4 to 1.2 μm is shown in Fig. 3(b). The degree to which a photovoltaic cell will make carriers from the impinged photons of a specific energy is indicated by its quantum efficiency. We find that at longer wavelengths, there is more photon absorption due to the increase in absorber thickness. Relatively longer wavelength photons i.e. low energy photons are absorbed at far distances from the WS_2_/Ag_3_CuS_2_ heterojunction. Therefore, photogenerated carriers increase within the absorber layer with increasing thickness causing the increase in QE in the longer wavelength [57]. In addition, light does not get absorbed lower energy photons, so QE decreases to zero at wavelengths greater than 1165 nm. For all results, a reduction of quantum efficiency is observed at wavelengths less than 550 nm. This is happened as a consequence of the absorption of light in WS_2_ window layer.

Fig. 3(c) depicts the responsivity versus wavelength for Ag_3_CuS_2_ layer widths ranging from 0.4 μm to 1.2 μm. For wavelength of 305–750 nm, the responsivity gradually increase to 0.6AW^-1^. After 750 nm, the responsivity increases to almost 0.79 AW^-1^ which referred near-IR region and falls to zero after 1165 nm.

Fig. 3(d) displays the Ag_3_CuS_2_ photodetector device's detectivity which has been measured for various absorber layer widths. As the absorber width increases, detectivity rises to a maximum of 4.73 × 10^14^ Jones for 0.8 μm width at a wavelength of 1065 nm. After that, it falls when the absorber width increases further. This can be explained by the interaction between reducing collection efficiency of photo-carriers with a considerable rise in absorber breadth and increasing QE with increasing absorber width [58].

#### 3.2.2 Role of absorber layer doping on Ag_3_CuS_2_ PD

**Fig. 4:**
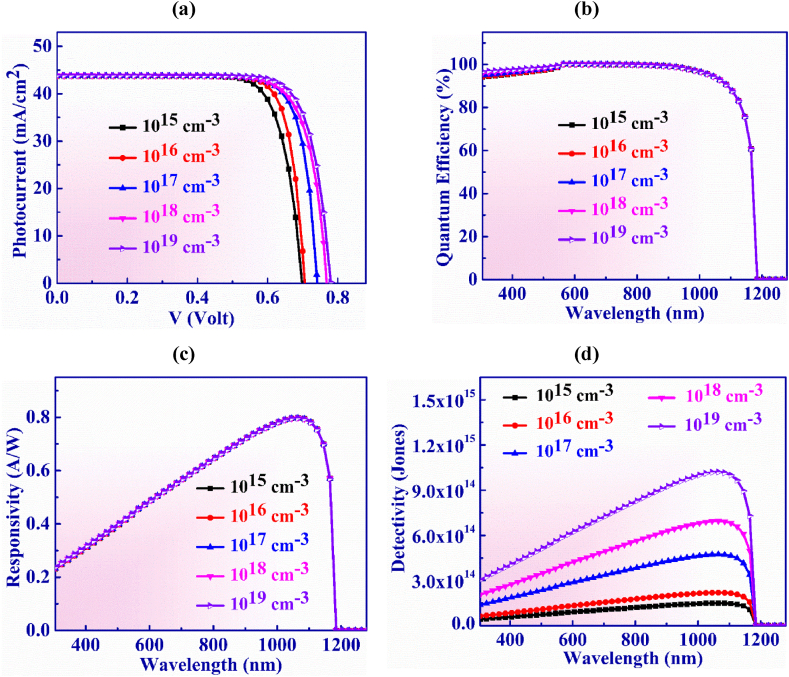
(a) Photocurrent vs. Voltage (b) QE, (c) Responsivity and (d) Detectivity characteristic curves of Ag_3_CuS_2_ photodetector with doping of Ag_3_CuS_2_ layer.

Ag_3_CuS_2_ layer carrier concentration is altered from 10^15^ to 10^19^ cm^−3^ in order to analyse the J-V characteristics of proposed structure as outlined in Fig. 4(a). It is evident that Voc rises with acceptor doping concentration from 0.68 to 0.78 V and Jsc menifests consistent behaviour with 43.75 mA/cm^2^, respectively. Higher doping causes an increase in built-in voltage which raises the Voc. That is happened because of the higher built-in-potential comes into existence at the absorber layer interface [52].

Fig. 4(b) illustrates the quantum efficiency vs wavelength for Ag_3_CuS_2_ layer doping concentration. It is found that QE exhibits consistent behavior and is unaffected by greater doping concentrations. Moreover, at wavelengths longer than 1165 nm, QE drops to zero since photons with energy less than that of the absorbed bandgap is not absorbed in the PD.

The responsivity exhibits consistent characteristics as seen in Fig. 4(c), just as QE does for all values of doping concentration. The greatest value of responsivity of 0.79 AW^-1^ is displayed at 1065 nm at a doping of 10^17^ cm^−3^.

Detectivity vs wavelength for Ag_3_CuS_2_ layer doping concentration is displayed in Fig. 4(d) with a variation from 10^15^ to 10^19^ cm^−3^. The detectivity increases from 1.5 × 10^14^ to 1 × 10^15^ Jones as the doping concentration is increased. This comes into being due to the fact that higher value of doping increases the V_OC_ of the device that specifies the reduction in dark current that leads to the increase in detectivity [53]. The optimized device shows a maximum detectivity of 4.73 × 10^14^ Jones at a wavelength of 1065 nm with doping concentration of 10^17^ cm^−3^.

#### 3.2.3 Mastery of defects of absorber layer on Ag_3_CuS_2_ PD

**Fig. 5:**
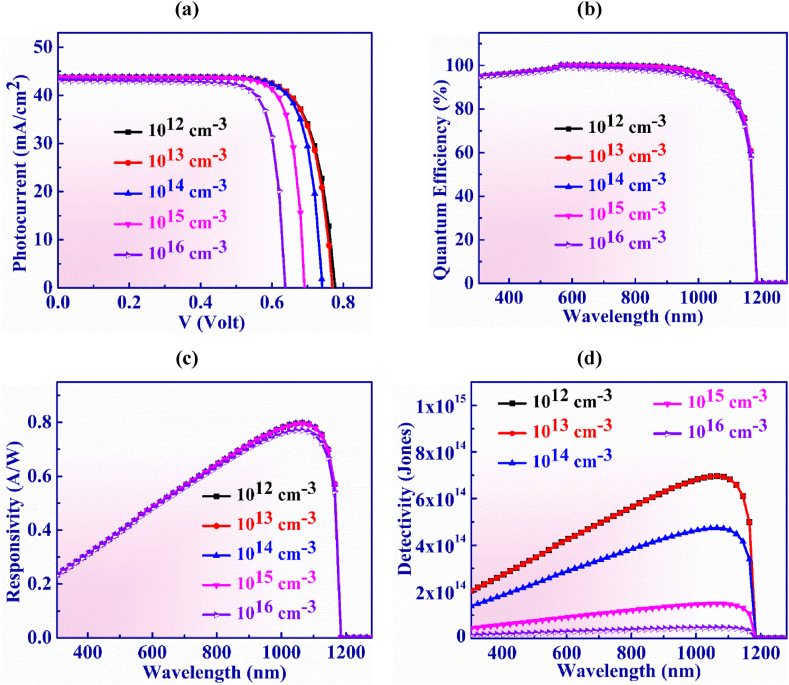
(a) Photocurrent vs. Voltage (b) QE, (c) Responsivity and (d) Detectivity characteristic curves of Ag_3_CuS_2_ based photodetector with defects in Ag_3_CuS_2_ layer.

The concentration of bulk defect in Ag_3_CuS_2_ layer is varied from 10^12^ cm^−3^ to 10^16^ cm^−3^ in order to analyse the J-V characteristics of the designed PD structure as illustrated in Fig. 5(a). Here, the J_SC_ shows about an unchanged behavior with little alteration from 43.8 to 43.04 mA/cm^2^ owing to the rise in defects. As the absorption of photons can be hampered by the larger amount defects and consequences the smaller rate of generating electron-hole pairs, the device current will under go a reduction owing to the escalation of recombination current. V_OC_ downfalls quickly by the rise in recombination current [59]. This phenomenon of the V_OC_ takes place due to the minority carrier lifetime and diffusion length are higher in this range of defect in the Ag_3_CuS_2_ PD [60]. Although photocurrent manifests a constant nature in the entire range of defect densities, the device voltage degrades successively as observed in the figure. For the volume defects of 10^16^ cm^−3^, V_OC_ abates from 0.76 to 0.62 V.

Fig. 5(b) illustrates the quantum efficiency vs wavelength for Ag_3_CuS_2_ layer defect concentration which is adjusted from 10^12^ to 10^16^ cm^−3^. It is discovered that QE exhibits consistent behavior and is unaffected by greater defect concentrations.

Detectivity vs. wavelength for Ag_3_CuS_2_ layer defect concentration is displayed in Fig. 5(d) with a variation from 10^12^ to 10^16^ cm^−3^. The detectivity decreases from 6.96 × 10^14^ to 4.59 × 10^13^ Jones as the defect concentration is increased. This is due to the fact that recombination rises as defect concentration rises, leading to a rise in dark current [53]. Detectivity falls when dark current increases. The optimized Ag_3_CuS_2_ PD device shows a maximum detectivity of 4.73 × 10^14^ Jones at a wavelength of 1065 nm with defect concentration of 10^14^ cm^−3^.

### 3.3 Effect of WS_2_ buffer layer

#### 3.3.1 Impact of WS_2_ layer thickness on Ag_3_CuS_2_ photodetector

**Fig. 6:**
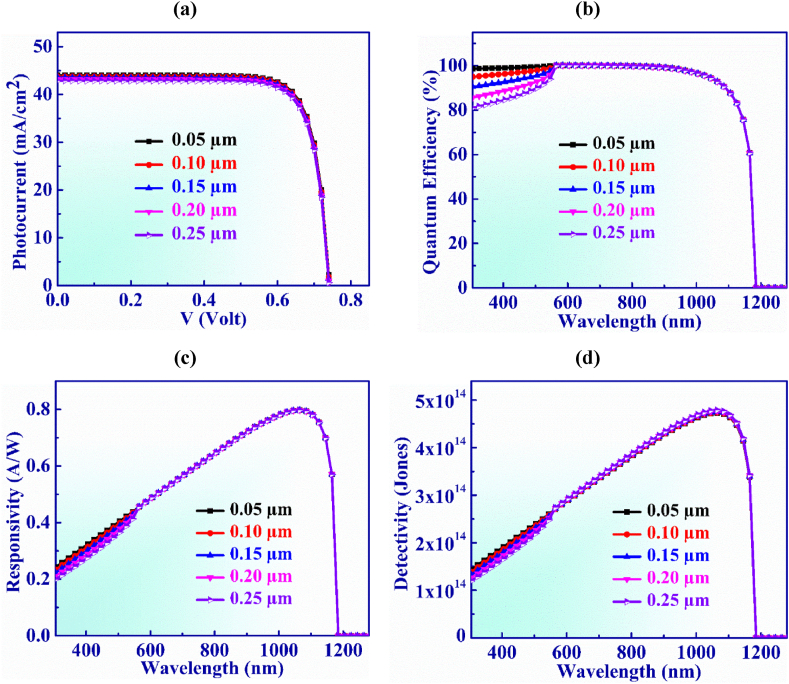
(a) Photocurrent vs. Voltage (b) QE, (c) Responsivity and (d) Detectivity characteristic curves of Ag_3_CuS_2_ photodetector with WS_2_ layer width.

Fig. 6(a) shows the dominance of the variation of WS_2_ window on the operation of Ag_3_CuS_2_ PD. The width of the WS_2_ layer fluctuates between 0.05 and 0.25 μm. Although, the V_OC_ remains constant at 0.74 V but J_SC_ lessens from 43.99 to 42.87 mA/cm^2^ within this thickness range. This is because the thickness of the window (WS_2_) layer increases parasitic absorption which perturbs photons at higher wavelengths so they cannot reach the absorber layer and reduces output current [61].

Fig. 6(b) illustrates the quantum efficiency vs. wavelength for WS_2_ layer width which is adjusted from 0.05 to 0.25 μm. It is visualized from the figure that QE exhibits constant behavior and is unaffected by higher thickness values above 0.25 μm. Additionally, at wavelengths longer than 1165 nm, QE drops to zero. However, at the shorter wavelength, the QE drops when the thickness of the WS_2_ layer rises. This phenomenon occurs due to absorption of light in the window layer which could be called window gain [62]. Moreover, the decrease in quantum efficiency for shorter wavelengths with increased thickness of the window layer is attributed to increase in recombination in the WS_2_ layer as higher thickness absorbs more photons that reduces J_SC_ as well as QE of the devices [63].

The responsivity exhibits consistent characteristics as depicted in Fig. 6(c). The greatest value of responsivity of 0.79 AW^-1^ is displayed at 1065 nm wavelength of light when the width of WS_2_ window is 0.1 μm.

Detectivity vs. wavelength for WS_2_ layer thickness is displayed in Fig. 6(d) within the range of 0.05–0.25 μm. The detectivity insignificantly increases from 4.72 × 10^14^ to 4.78 × 10^14^ Jones with the increasing of thickness. The detectivity shows an almost constant value for the width of the buffer layer in the Jalpaite PD.

#### 3.3.2 Impact of WS_2_ layer doping on Ag_3_CuS_2_ photodetector

**Fig. 7:**
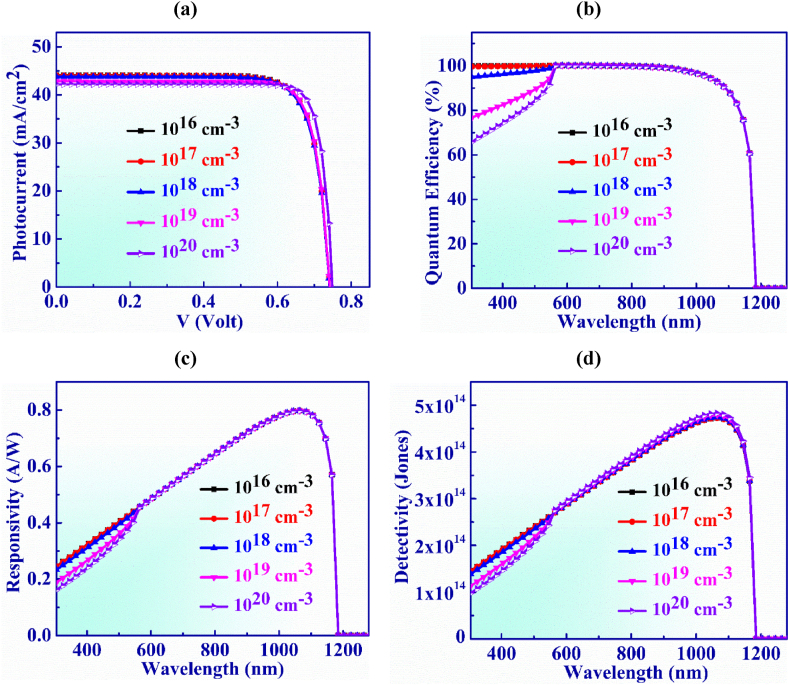
(a) Photocurrent vs. Voltage (b) QE, (c) Responsivity and (d) Detectivity characteristic curves of Ag_3_CuS_2_ based photodetector with WS_2_ layer doping.

Fig. 7(a) delineates the role of doping concentration of the WS_2_ layer on the functioning of Ag_3_CuS_2_ PD. WS_2_ layer doping, N_D_ is changed from 10^16^ to 10^20^ cm^−3^ in order to investigate the J-V characteristics of the PD structure. In this range, the J_SC_ is slightly decreased from 44.06 to 42.18 mA/cm^2^ due to increasing of parasitic absorption which means higher recombination rate of electron-hole pairs (EHPs) and the V_OC_ shows a slight increase from 0.74 to 0.75 V following the doping [52]. This kind of situation arises because doping raises intrinsic potential which lowers dark current [59].

Fig. 7(b) illustrates the quantum efficiency vs. wavelength for WS_2_ layer doping concentration which is adjusted from 10^16^ to 10^20^ cm^−3^. It is observed that QE exhibits consistent behavior and is unaffected by greater doping concentrations. For N_D_ values of 10^16^ and 10^17^cm^−3^, the QE is 100 % for wavelength of 550–750 nm. Notably, for shorter wavelengths, the quantum efficiency is decreased with the increased of doping level. This is happened because the higher donor can increase the recombination current in the window layer that reduces J_SC_ and QE [63,64]. In an addition to this, at wavelengths longer than 1165 nm, QE drops to nil.

The responsivity exhibits consistent characteristics as delimitated in Fig. 7(c), just as QE does for all values of doping of WS_2_ layer. The greatest value of 0.79 AW^-1^ for responsivity is displayed at 1065 nm at N_D_ of 10^18^ cm^−3^.

Detectivity vs wavelength for WS_2_ layer doping concentration is displayed in Fig. 7(d). The detectivity marginaly increases from 4.72 × 10^14^ to 4.83 × 10^14^ Jones as the doping, N_D_ is uprised due to the climb in V_OC_ which indicates the fall in dark current with doping [53]. The detectivity remains at almost constant value for the doping of the buffer layer in the Jalpaite PD.

#### 3.3.3 Impact of WS_2_ layer defect on Ag_3_CuS_2_ PD

**Fig. 8:**
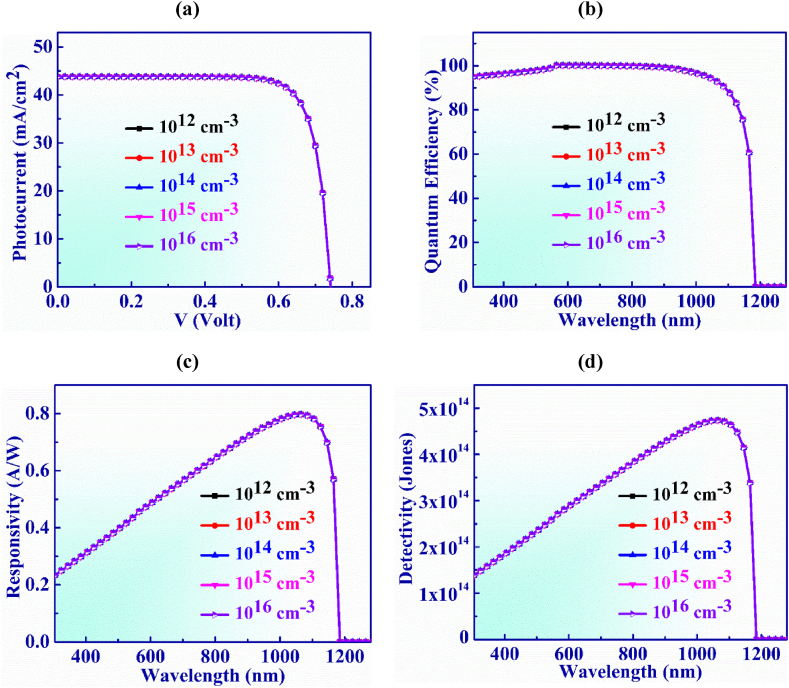
(a) Photocurrent vs Voltage (b) QE, (c) Responsivity and (d) Detectivity characteristic curves of Ag_3_CuS_2_ based photodetector with WS_2_ layer defects.

Fig. 8(a) expresses the variation in J-V characteristics with WS_2_ layer defects that have been varied from 10^12^ to 10^16^ cm^−3^ to analyse the PD structure. Here, the J_SC_ and V_OC_ have shown constant behavior with a value of 43.79 mA/cm^2^ and 0.74 V, respectively with the increase in bulk defects of WS_2_ buffer layer.

Fig. 8(b) illustrates the quantum efficiency vs. wavelength for WS_2_ layer defect concentration in the span of 10^12^ to 10^16^ cm^−3^. It is noticed that QE exhibits consistent behavior and is unaffected by defect concentrations.

The responsivity exhibits consistent characteristics as seen in Fig. 8(c). The greatest value of 0.79 AW^-1^ for responsivity is displayed at 1065 nm with defect concentration of 10^14^ cm^−3^ of this WS_2_ buffer layer.

Detectivity vs. wavelength for WS_2_ layer defect concentration is displayed in Fig. 8(d) with a variation from 10^12^ to 10^16^ cm^−3^. The detectivity also shows a consistant behavior with a value of 4.73 × 10^14^ Jones as photocurrent and volatge remain unchanged with defects of WS_2_ layer.

### 3.4 Effect of BaSi_2_ BSF layer

#### 3.4.1 Impact of BaSi_2_ BSF layer thickness on Ag_3_CuS_2_ photodetector

**Fig. 9:**
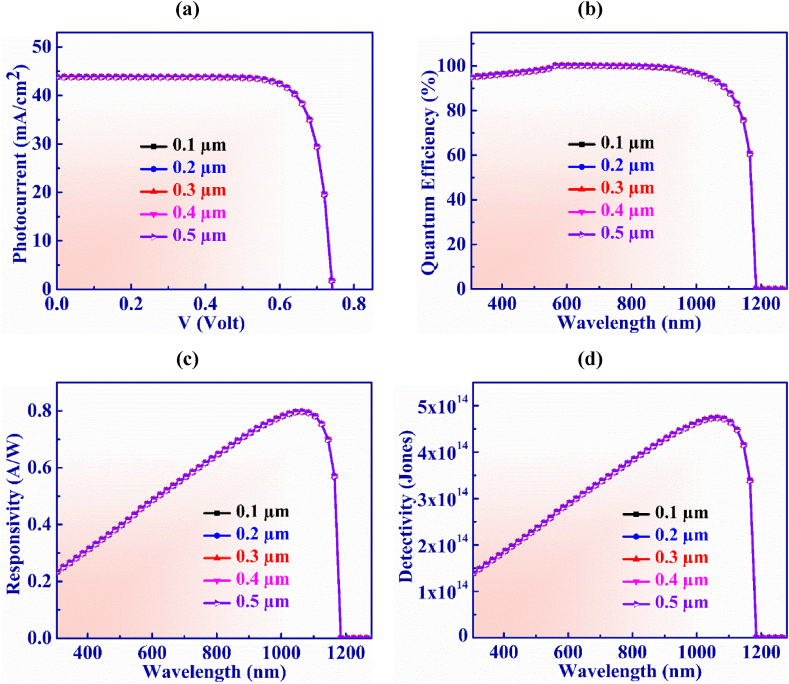
(a) Photocurrent vs. Voltage, (b) QE, (c) Responsivity and (d) Detectivity characteristic curves of Ag_3_CuS_2_ based photodetector with BaSi_2_ layer thickness.

Fig. 9(a) shows that the fluctuation in J-V curves with width of the BaSi_2_ layer between the range from 0.1 to 0.5 μm. V_OC_ and J_SC_ shows constant behavior with the BSF thickness variation at 0.74 V and 43.8 mA/cm^2^, respectively.

Fig. 9(b) illustrates the quantum efficiency vs wavelength for BaSi_2_ layer thickness which is changed from 0.1 to 0.5 μm. QE exhibits consistent behavior and is unaffected at greater thickness as seen in the specific figure. The responsivity exhibits consistent characteristics as seen in Fig. 9(c). The greatest value of 0.79AW^-1^ for responsivity is displayed at 1065 nm at 0.2 μm width of BaSi_2_ BSF layer.

The change in detectivity with BaSi_2_ layer thickness is displayed in Fig. 9(d) with a span from 0.1 to 0.5 μm. The detectivity show consistant behaviour with the increasing of thickness. The gratest value of detectivity obtained is 4.73 × 10^14^ Jones at 1065 nm with 0.2 μm width of BaSi_2_ layer.

#### 3.4.2 Impact of BaSi_2_ BSF doping on Ag_3_CuS_2_ PD

**Fig. 10:**
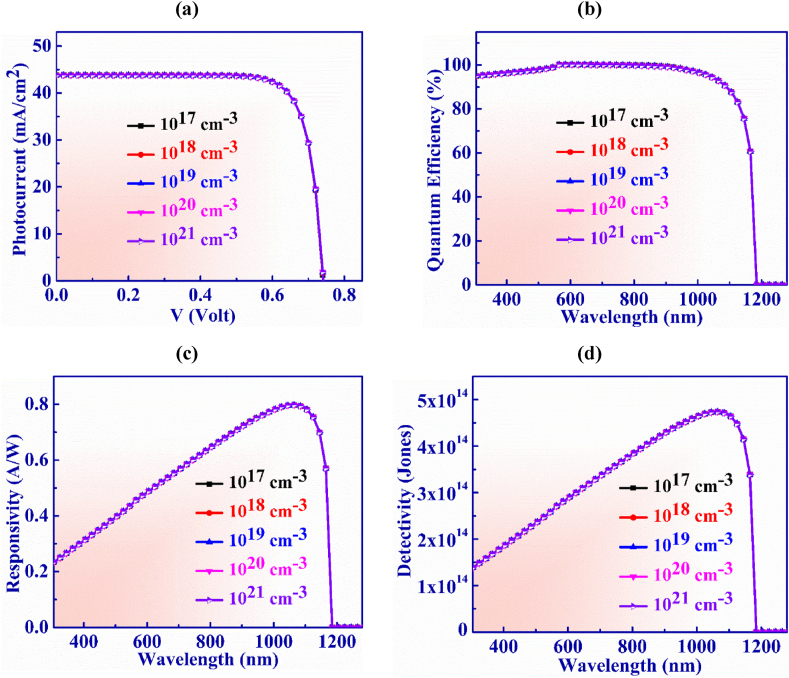
(a) Photocurrent vs. Voltage (b) QE, (c) Responsivity and (d) Detectivity characteristic curves of Ag_3_CuS_2_ based photodetector with doping of BaSi_2_ layer.

Fig. 10(a) depicts the impression of doping in the BaSi_2_ layer on the operation of the designed PD. Doping concentration is changed from 10^17^ cm^−3^ to 10^21^ cm^−3^ in order to examine the J-V characteristics of the suggested structure. In this range, the V_OC_ and J_SC_ shows constant behavior with the doping concentration at 0.74 V and 43.8 mA/cm^2^, respectively.

Fig. 10(b) illustrates the quantum efficiency vs. wavelength for WS_2_ layer doping concentration which is varied from 10^17^ to 10^21^ cm^−3^. It is noticed that QE exhibits consistent behavior and is unaffected by greater doping concentrations.

The responsivity exhibits consistent characteristics just as QE does as seen in Fig. 10(c). The highest value of 0.79 AW^-1^ for responsivity is displayed at 1065 nm at doping of 10^19^ cm^−3^.

Detectivity variation with BaSi_2_ layer doping concentration is displayed in Fig. 10(d) in the doping range from 10^17^ to 10^21^ cm^−3^. The detectivity shows consistant behaviour with the increasing of doping concentration. The gratest value of detectivity is 4.73 × 10^14^ Jones at 1065 nm obtained with doping concentration of 10^19^ cm^−3^.

#### 3.4.3 Influence of BaSi_2_ BSF defects on Ag_3_CuS_2_ PD

**Fig. 11:**
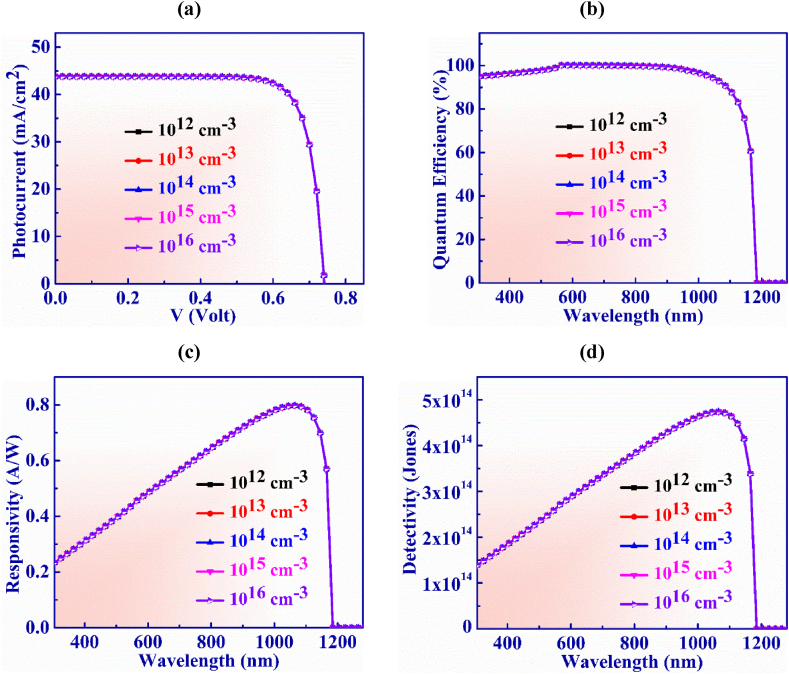
(a) Photocurrent vs. Voltage (b) QE, (c) Responsivity and (d) Detectivity characteristic curve of Ag_3_CuS_2_ based photodetector with BaSi_2_ layer defects.

Defect concentration of the BaSi_2_ layer is changed from 10^12^ cm^−3^ to 10^16^ cm^−3^ in order to examine the J-V properties of the suggested PD structure as seen in Fig. 11(a). In this range, the V_OC_ and J_SC_ show constant behavior at 0.74 V and 43.8 mA/cm^2^, respectively with the defect concentration.

Fig. 11(b) illustrates the quantum efficiency vs. wavelength for WS_2_ layer defect concentration which is altered from 10^12^ to 10^16^ cm^−3^. It is visualized that QE exhibits steady behavior and is unaffected by greater defect concentrations.

The responsivity exhibits consistent characteristics just as QE does with defects as seen in Fig. 11(c) The highest values of 0.79 AW^-1^ for responsivity is displayed at 1065 nm with defects of 10^14^ cm^−3^.

Detectivity variation with BaSi_2_ BSF layer's doping concentration is displayed in Fig. 11(d). The variation of doping concentration is observed at the range of 10^17^ to 10^21^ cm^−3^. The detectivity shows steady behaviour with the increasing of defect concentration. The gratest value of detectivity obtained is 4.73 × 10^14^ Jones at 1065 nm with defect concentration of 10^14^ cm^−3^.

### 3.5 Impression of resistances on Ag_3_CuS_2_ PD

**Fig. 12:**
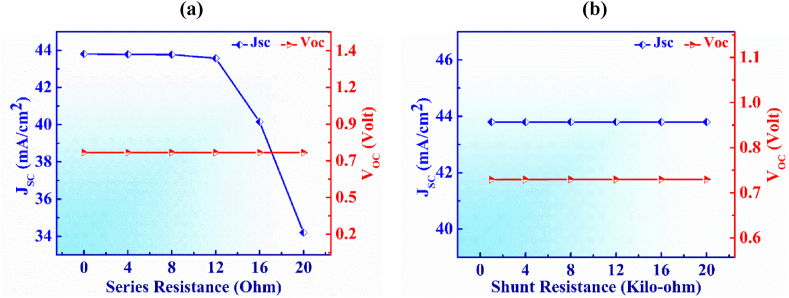
Variation in photocurrent and Voltage with (a) series and (b) shunt resistances of Ag_3_CuS_2_ based photodetector.

Fig. 12 (a) and (b) show the fluctuations of J_SC_ and V_OC_ with the variations of series and shunt resistances of the PD device, respectively. The body and contacts of a cell provide the series resistance, but various fabrication flaws that cause leakage current at the junctions are in charge of the shunt resistance [65]. The variation of J_SC_ and V_OC_ in the range of 0–20 Ω cm^2^ is depicted in Fig. 12(a) along with the variation of series resistance. It can be observed that the V_OC_ stay constant at 0.74 V and J_SC_ constant until 12 Ω cm^2^, then dropping from 43.7 to 34.2 mA/cm^2^ with additional series resistance. On the other hand, the device's shunt resistance ranges from 1 to 20 kΩ cm^2^ shown in Fig. 11(b). The figure describes how both V_OC_ and J_SC_ exhibit continuous behaviour at 0.74 V and 43.7 mA/cm^2^ with shunt resistance.

### 3.6 Overall photodetector performances

The photodetector's overall performance, both with and without a BSF layer is displayed in Table 2 based on the peak performance value of Ag_3_CuS_2_ jalpaite PD.

**Table 2:** The optimized perfornace parameters of Ag_3_CuS_2_ jalpaite photodetector.

Device Structure	Photocurrent (mA/cm^2^)	Voltage (Volt)	Responsivity, R (A/W)	Detectivity, D* (Jones)
n-WS_2_/p-Ag_3_CuS_2_	43.4	0.66	0.78	1 × 10^14^
n-WS_2_/p-Ag_3_CuS_2_/p^+^-BaSi_2_	43.79	0.74	0.79	4.73 × 10^14^

## 4 Conclusion

This study reports on the performance examination of the Ag_3_CuS_2_ based photodetector for a variety of electrical and optical characteristics, including quantum efficiency, open circuit voltage, responsivity, and detectivity. The photodetector provides a short circuit current of J_SC_ = 43.79 mA/cm^2^, responsivity of R = 0.79 AW^-1^, and detectivity of D* = 4.73 × 10^14^ Jones, according to the simulation results. The Ag_3_CuS_2_ PD exhibits significant promise as a potential replacement for other heterojunction-based photodetectors. The results prove this material as a futuristic one to detect NIR wavelengths within a single framework and unveil the potentiality of this newly jalpaite. Its remarkable capability positions it as a favorable one for practical applications.

## Data availability

Data will be available from the corresponding author upon reasonable request.

## Declaration of generative AI and AI-assisted technologies

The authors did not use AI or AI-assisted technologies during the preparation fo this manuscript.

## CRediT authorship contribution statement

**Sheikh Noman Shiddique:** Writing – original draft, Validation, Methodology, Investigation, Formal analysis, Data curation. **Md. Islahur Rahman Ebon:** Writing – original draft, Validation, Investigation, Formal analysis. **Md. Alamin Hossain Pappu:** Writing – original draft, Validation, Investigation, Formal analysis. **Md. Choyon Islam:** Writing – original draft, Formal analysis, Data curation. **Jaker Hossain:** Writing – review & editing, Writing – original draft, Visualization, Validation, Supervision, Investigation, Formal analysis, Conceptualization.

## Declaration of competing interest

The authors declare that they have no known competing financial interests or personal relationships that could have appeared to influence the work reported in this paper.
